# A surgical resection case of myxoma arising from the posterior wall of the left atrium complicated with complete atrioventricular block

**DOI:** 10.1186/s13019-024-02715-w

**Published:** 2024-04-16

**Authors:** Satoshi Sakakibara, Takashi Yamauchi, Takahiro Ohmori

**Affiliations:** Department of Cardiovascular Surgery, Higashiosaka City Medical Center, 3-4-5 Nishi iwata, Higashiosaka, 578-8588 Osaka Japan

**Keywords:** Myxoma, Atrioventricular block, Left atrium, Posterior wall

## Abstract

An 80-year-old female was referred to our institution due to transient right upper limb weakness. Transthoracic and transesophageal echocardiography revealed a tumor in the left atrium. The tumor was attached to the posterior wall of the left atrium near the atrioventricular node. Intraoperative pathological examination revealed that the tumor was a myxoma, and complete resection was successfully performed. However, she experienced persistent complete atrioventricular block postoperatively and required pacemaker implantation.

## Introduction

Myxomas usually originate from the left atrium [1], and it is generally attached to the fossa ovalis of the interatrial septum [2]. Myxomas arising from the posterior wall of the left atrium (LA) are rare. Herein, we report a case of surgical resection of a myxoma arising from the posterior wall of the LA near the atrioventricular (AV) node, complicated by a complete atrioventricular block (CAVB) postoperatively.

## Case report

**Fig. 1:**
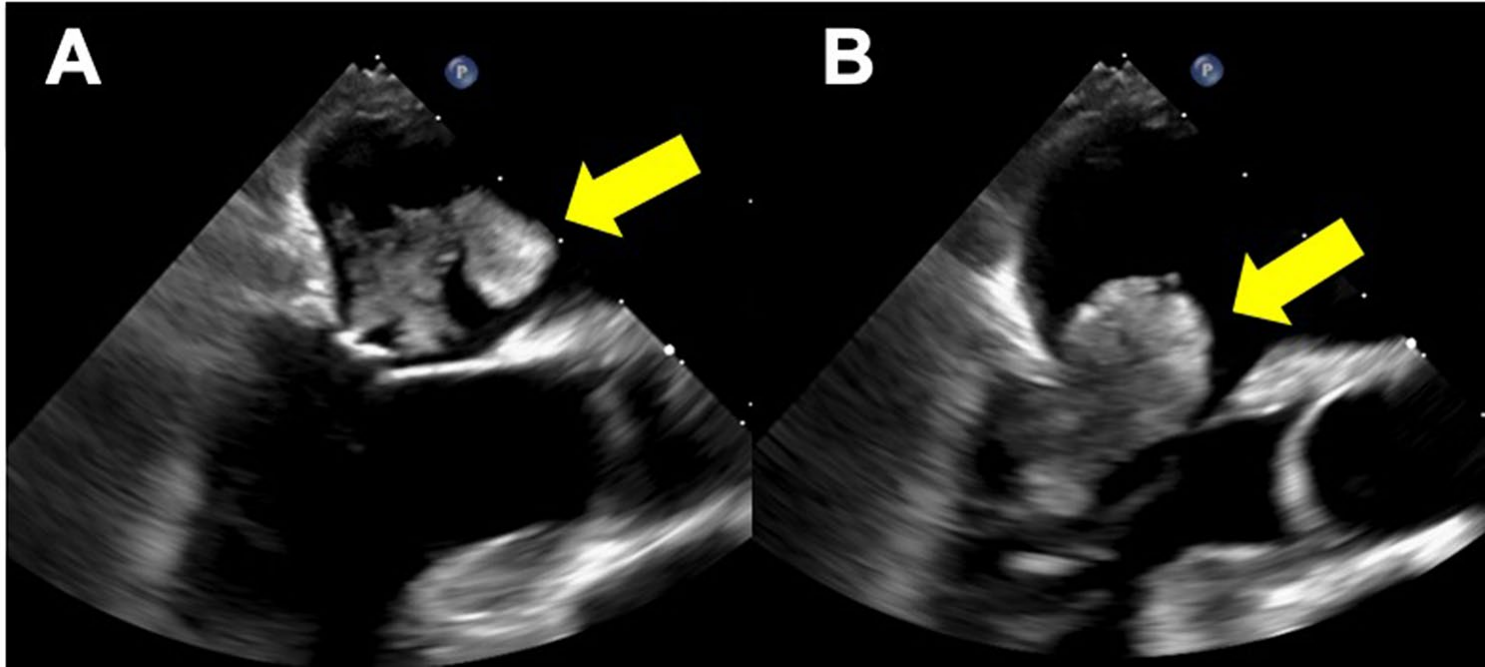
Preoperative transesophageal echocardiography. The left atrial tumor (yellow arrow) was attached to the anterior mitral leaflet. No significant mitral regurgitation was observed (**A**, **B**)

**Fig. 2:**
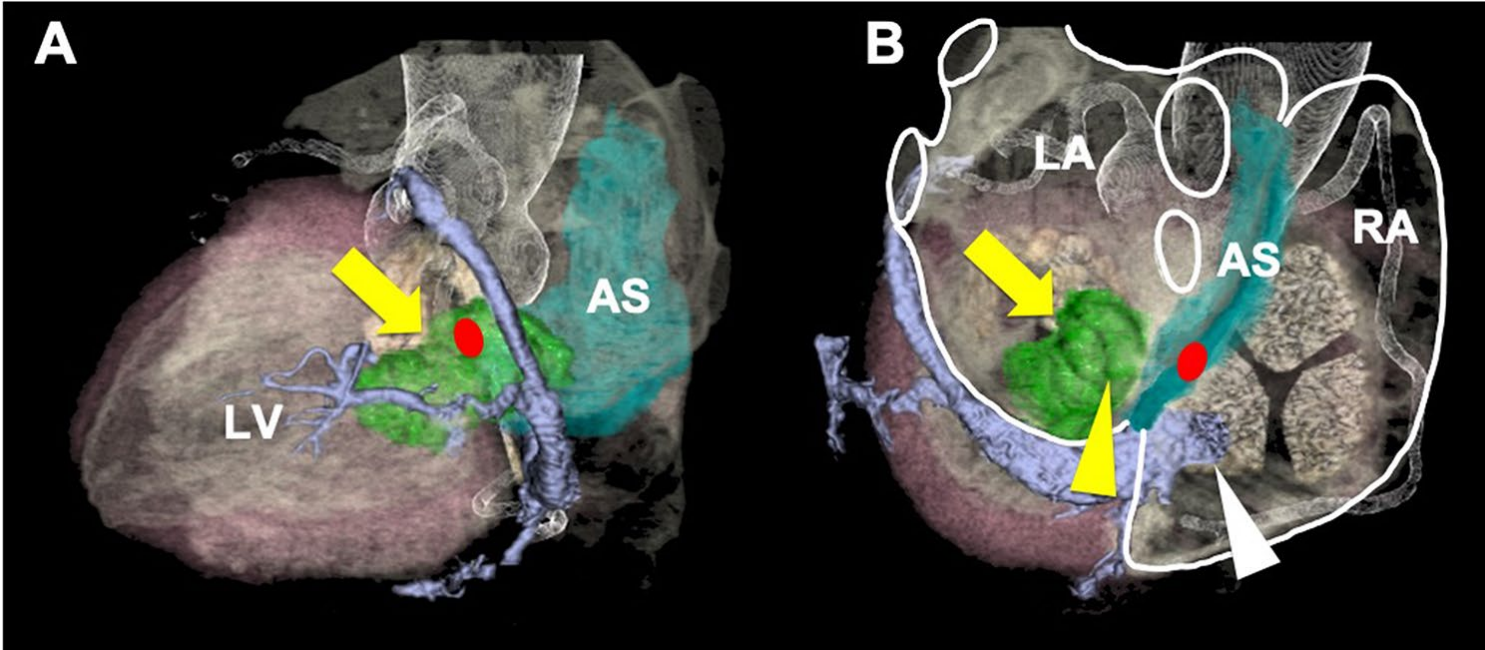
Cardiac CT. The left atrial tumor (yellow arrow) had no significant nutrient vessels. Its stalk was attached to the posterior wall of LA (yellow arrowhead). This tumor is close to the coronary sinus and its ostium (white arrowhead) and atrioventricular node (red circle). CT: computed tomography, LV: left ventricle, AS: atrial septum, LA: left atrium, RA: right atrium

An 80-year-old female with transient right upper limb weakness was referred to our institution. Her medical history included hypertension, dyslipidemia, and type 2 diabetes mellitus, all of which were treated with oral medications. Physical examination upon admission revealed a regular pulse of 78 beats/min and a blood pressure of 155/101 mmHg. Blood tests revealed no significant findings and well-controlled dyslipidemia (low-density lipoprotein cholesterol, 67 mg/dL) and diabetes mellitus (hemoglobin A1c 6.2%). Brain magnetic resonance imaging revealed an acute cerebral infarction in the left precentral gyrus. Transthoracic echocardiography (TTE) revealed a tumor that was 20 × 33 mm in the LA. The tumor floated into the left ventricle through the mitral valve orifice during diastole. TTE also showed no significant mitral regurgitation (MR) or mitral stenosis (MS) and a left ventricular ejection fraction of 72%. Transesophageal echocardiography (TEE) revealed a left atrial tumor attached to the anterior mitral leaflet, with no significant MR or MS (Fig. 1). Cardiac computed tomography revealed a left atrial tumor attached to the posterior wall of the LA, close to the coronary sinus (Fig. 2), and no substantial coronary artery stenosis. However, tumor nutrient vessels were not detected. We considered this patient to be at risk of embolization and sudden death. Therefore, we planned a cardiac tumor resection four days after diagnosing the patient with acute cerebral infarction.

**Fig. 3:**
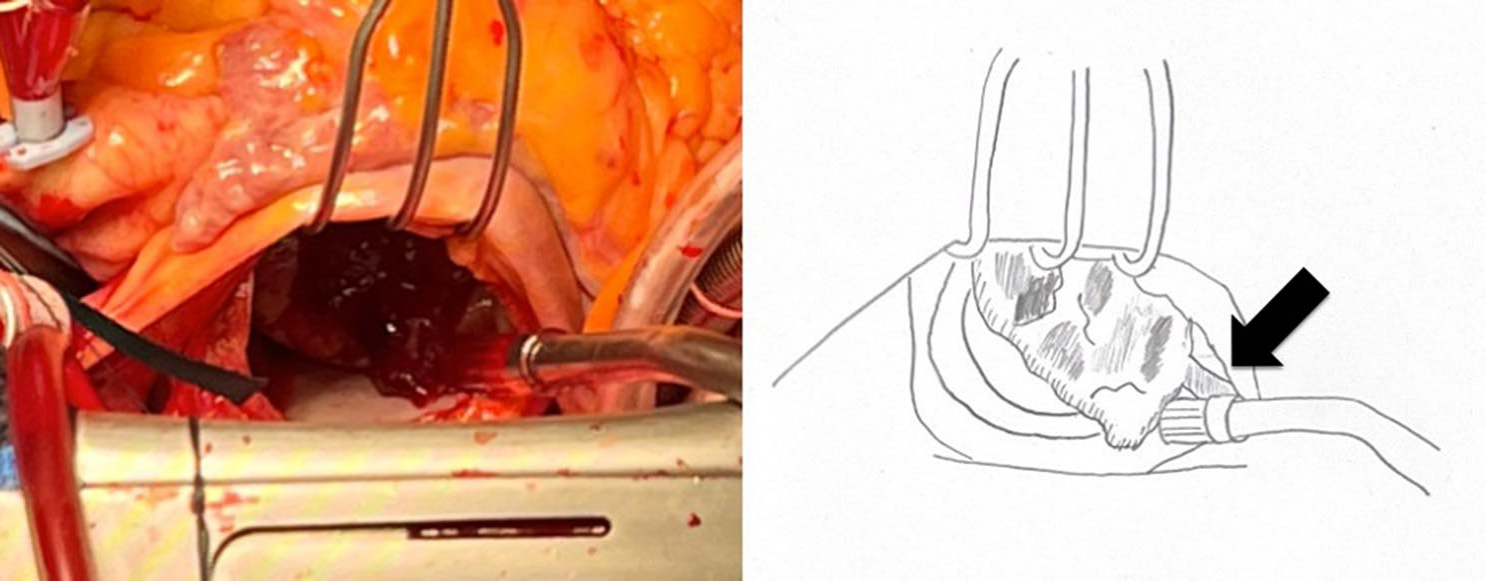
Intraoperative findings. The tumor was dark red, jelly-like, and approximately 30 mm in size. It was attached to the posterior wall of the LA (black arrow). LA: left atrium

**Fig. 4:**
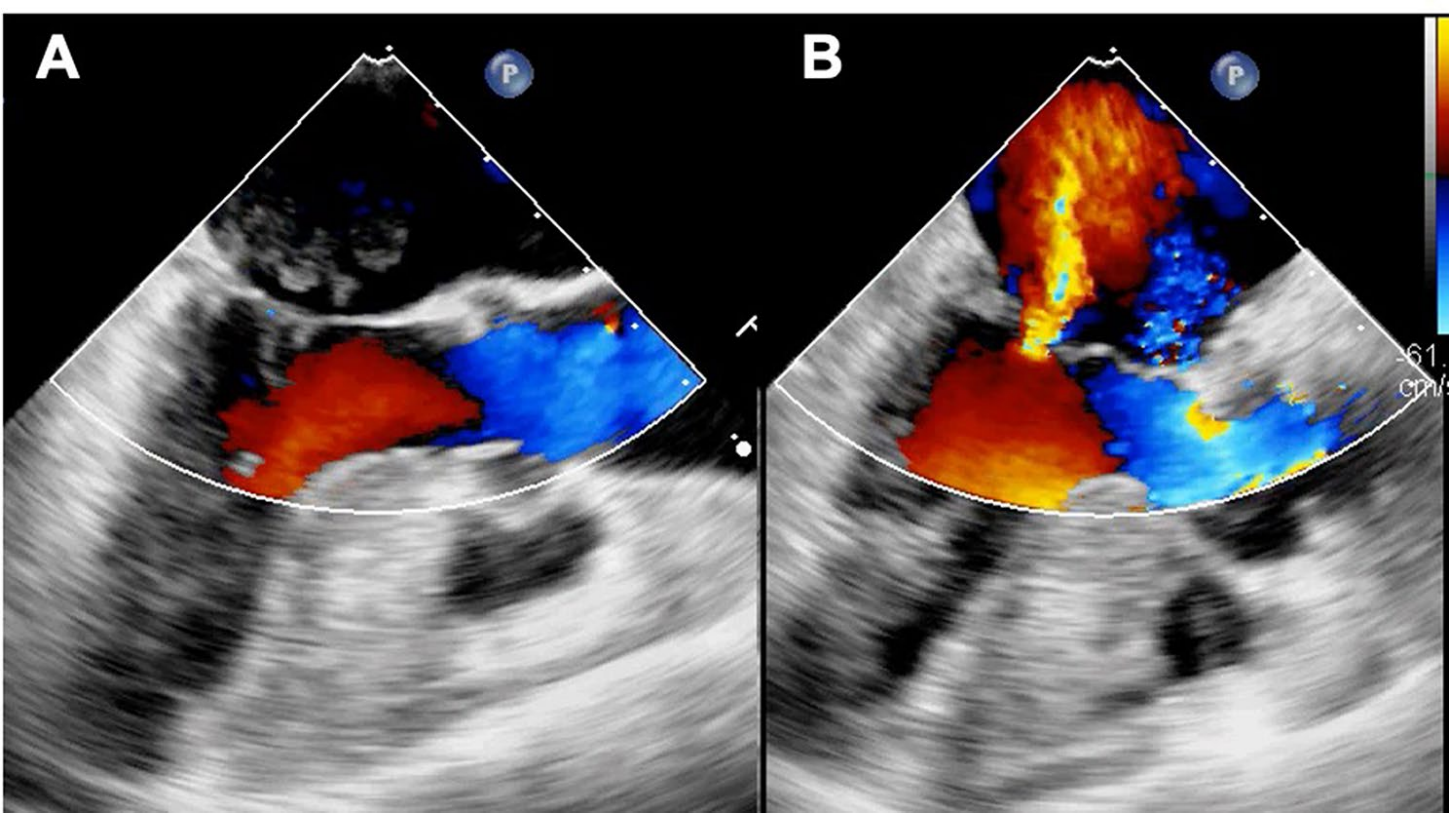
Intraoperative transesophageal echocardiography. Before tumor resection, no significant MR was detected (**A**). After tumor resection, severe MR due to A2 prolapse was demonstrated for the first time (**B**). MR: mitral regurgitation

General anesthesia was induced. Subsequently, a median sternotomy was performed, and cardiopulmonary bypass (CPB) was established via aortic and bicaval cannulations. The aorta was cross-clamped, and the heart was arrested with antegrade cardioplegia. The LA was examined using a right-sided left-atrial approach. The tumor was observed to be dark red, jelly-like, and approximately 30 mm in size. Its stalk was attached to the posterior wall of the LA, close to the AV node and orifice of the coronary sinus (Fig. 3). Therefore, an atrial septal incision was performed. The tumor was delicate and had to be resected into pieces. Special care was taken to avoid damage to the coronary sinus and AV node, as the stalk was resected with a 5 mm margin around its base. The atrial septum and LA were closed directly. After declamping the ascending aorta, the patient had a CAVB. Intraoperative TEE demonstrated severe mitral regurgitation due to A2 prolapse (Fig. 4). Myxomas have the potential to mask mitral valve prolapse. Consequently, cardiac arrest was performed again, and mitral valve repair was conducted with artificial chordae reconstruction, 28-mm Sorin-MEMO 3D annuloplasty ring (Sorin Group Italia S.r.L., Saluggia, Italy), and P1/P2 indent closure. A saline test revealed no significant MR. The patient was easily weaned from CPB, and protamine was systemically administered. As her heart rhythm was CAVB, temporary pacing was necessary.

**Fig. 5:**
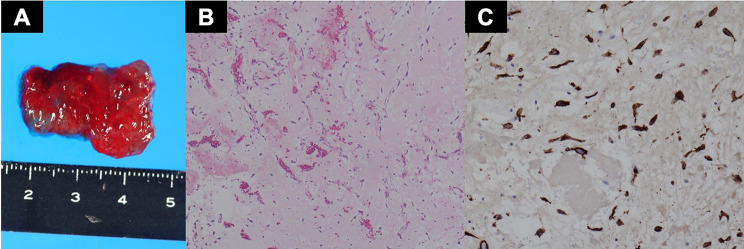
Histological examination. The resected specimen was observed to be dark red and jelly-like (**A**). The tumor was composed of stellate and spindled cells (**B**, hematoxylin & eosin, ×40). Tumor cells were CD34 positive (**C**, immunohistological staining of CD34, ×100)

Postoperative histopathological analysis revealed that the tumor was a myxoma with no tumor cells in the resected margin (Fig. 5), and TTE showed no evidence of residual tumor, trivial MR, or significant atrial shunt flow. Pacemaker implantation was performed for the CAVB on the 11th postoperative day, and the patient was discharged on the 21st postoperative day.

## Discussion

Myxomas generally arise in the left atrium, with approximately 75% of cases occurring there, while 15–20% occur in the right atrium and the remainder in the ventricle [1]. Most left atrial myxomas originate from the fossa ovalis. Left atrial myxomas rarely arise from the anterior wall of the LA or the left atrial appendage [2]. Pinede et al. reported that myxomas originating from the posterior wall of the LA account for approximately 5% of left atrial myxoma [3].

The treatment of myxoma involves surgical resection to prevent embolization, ischemic attack, and sudden death caused by prolapse into the left ventricle across the mitral valve. Atrial myxomas are usually resected along the atrial wall to prevent recurrence [4]. The postoperative recurrence rate is estimated to be 5% [3, 5]. However, in the case of myxoma arising from the posterior wall of the LA, resection of the atrial wall may be impossible to avoid damage to the surrounding organs, such as the esophagus [2]. Mochizuki et al. [2] reported that, if a myxoma cannot be completely resected, myxoma recurrence can be prevented by removing the tumor’s nutrient vessels. Such removal can prevent heart failure caused by shunt flow and coronary stealing syndrome. Therefore, the tumor’s nutrient vessels must be located via computed tomography or coronary angiography [2]. In addition, they also reported that the superior transseptal approach, which involves incising the upper left atrial wall, can be used to expand the entire left atrial lumen and is useful in cases of multiple tumors or cases in which the tumors are attached to the posterior wall of the LA. However, because the sinoatrial nodal artery extends along the superior wall of the LA, further extension of the left superior wall incision should be considered for postoperative sick sinus syndrome [2].

In this case, the myxoma was attached to the posterior wall of the LA and the tumor with its stalk was resected completely using a right-sided left atrial approach. The CAVB persisted postoperatively, and pacemaker implantation was required. It is important to consider that some cases of complete surgical resection of left atrial myxoma may be complicated by CAVB, especially in cases originating from the left atrial posterior wall near the AV node. Furthermore, if a left atrial myxoma floats into the left ventricle through the mitral valve orifice, considering the possibility that the MR is masked preoperatively is crucial. This patient’s tendinous cords were intact. Preoperative TTE revealed no MS or left atrial enlargement. Therefore, we ruled out mitral annular enlargement caused by these factors, and we suspected degeneration of the valve leaflets themselves. Whether degeneration of the valve leaflets was causally related to the myxoma could not be determined.

## Conclusion

This report describes a case of tumor resection for a myxoma originating from the posterior wall of the LA. If a myxoma arises from the posterior wall of the LA, it is necessary to identify the location of tumor precisely, determine the extent of the resection site and take care of postoperative complications caused by the surrounding tissue damage including AV node. In addition, if a left atrial myxoma floats into the left ventricle through the mitral valve orifice, it is important to consider the possibility that the MR is masked preoperatively.

## Data Availability

No datasets were generated or analysed during the current study.
